# Self-Regulation Mediates the Relationship Between Stress and Quality of Life in Shift-Working Healthcare Professionals: Behavioral Clustering Insights

**DOI:** 10.3390/ejihpe15090180

**Published:** 2025-09-06

**Authors:** Mohammed F. Salahuddin, Jessica Walker, Ernesto Hernandez Zambrana, Vibhuti Gupta, Kwanghee Jung, Seithikurippu R. Pandi-Perumal, Md Dilshad Manzar

**Affiliations:** 1 Department of Pharmaceutical Sciences, School of Pharmacy & Health Professions, Notre Dame of Maryland University, Baltimore, MD 21210, USA; 2 Department of Computer Science and Data Science, School of Applied Computational Sciences, Meharry Medical College, Nashville, TN 37208, USA; 3 Department of Educational Psychology, Leadership, and Counseling, College of Education, Texas Tech University, Lubbock, TX 79409, USA; 4 Centre for Research and Development, Chandigarh University, Mohali 140413, Punjab, India; 5 Division of Research and Development, Lovely Professional University, Phagwara 144411, Punjab, India; 6 Department of Nursing, College of Applied Medical Sciences, Majmaah University, Majmaah 11952, Saudi Arabia

**Keywords:** self-regulation, perceived stress, quality of life, healthcare professionals, shift work, mediation analysis, burnout, behavioral clustering

## Abstract

The psychological mechanisms through which occupational stress impacts quality of life remain underexplored in shift-working healthcare professionals, a population exposed to unique stressors such as circadian disruption, high cognitive demands, and irregular work schedules. This study examined whether executive self-regulation mediates the relationship between perceived stress and quality of life in a sample of 82 shift-working healthcare professionals. Participants completed validated self-report measures, including the Perceived Stress Scale (PSS-4), Executive Skills Questionnaire–Revised (ESQ-R), and Quality of Life Scale (QOLS). Mediation analysis using 5126 bias-corrected bootstrapped samples revealed that perceived stress significantly predicted self-regulation difficulties, which in turn were associated with diminished quality of life. Self-regulation demonstrated an indirect-only mediation effect in both directions, though the forward path (stress → self-regulation → QOL) showed a stronger effect (indirect effect = −0.79; 95% CI: −1.63, −0.17), compared to the reverse path (QOL → self-regulation → stress; indirect effect = −0.04; 95% CI: −0.08, −0.01). Unsupervised K-means clustering identified three distinct behavioral clusters: resilient, low-strain, and high-strain, providing further support for personalized targeted interventions. These findings highlight self-regulation as a central mechanism through which stress affects quality of life and underscore the need for interventions that strengthen executive functioning in shift-based healthcare settings.

## 1. Introduction

Healthcare professionals who work night or rotating shifts, such as pharmacists, nurses, and clinical trainees, face unique occupational stressors that extend beyond routine workplace demands (Ugwu et al., 2024). Factors include circadian misalignment, sleep disruption, and high cognitive load, all of which contribute to elevated stress levels and compromised well-being (Jehan et al., 2017; Khan et al., 2021; Walker et al., 2020). Among healthcare professionals particularly, the simultaneous demands of academic progression, clinical training, and shift work amplify these challenges and heighten vulnerability to burnout and impaired quality of life (Rech et al., 2022; Salahuddin et al., 2025a; Schommer et al., 2022). Despite growing awareness of stress-related burnout in healthcare, the psychological mechanisms that translate stress exposure into life dissatisfaction remain underexplored. This gap is increasingly relevant as digital monitoring systems are being explored to model psychological strain and guide responsive health intervention (Lee et al., 2018; Mohr et al., 2017).

One such mechanism may be self-regulation, a multifaceted capacity encompassing emotional regulation, goal setting, impulse control, and time management (Baumeister et al., 2007; Billore et al., 2023; Dawson & Guare, 2016). Theoretically, self-regulation functions as a top-down system that helps individuals navigate complex demands, override short-term impulses, and align behavior with long-term goals (Gross, 1998; Heatherton, 2011). In high-stakes environments like healthcare, this capacity is critical for maintaining professional performance and personal well-being, especially under conditions of chronic stress and disrupted routines (Kadović et al., 2022; Lin et al., 2025). Advances in digital phenotyping offer new pathways for quantifying regulatory traits and predicting mental health risk, enabling targeted interventions to support healthcare workers (Barnett et al., 2018; Huckvale et al., 2019). Additionally, this study explicitly examines a reverse mediation pathway, testing whether quality of life may influence stress through improved self-regulation, to clarify the bidirectional nature of these relationships. This interdisciplinary focus resonates with emerging priorities in health, psychology, and education to model and mitigate stress-related dysfunction through scalable, data-driven tools.

The Transactional Model of Stress and Coping (Lazarus & Folkman, 1984) posits that stress outcomes depend not only on the nature of external demands but also on the cognitive and emotional resources available to manage them. Similarly, Emotion Regulation Theory (Gross, 1998) emphasizes that adaptive regulation strategies, such as reappraisal and attentional control, can buffer against negative emotional outcomes. Expanding on these foundations, more recent frameworks such as the Multisystem Resilience Framework (Kalisch et al., 2015) and the Integrated Model of Occupational Stress and Health (Bakker & Demerouti, 2017) suggest that cognitive-affective systems, including executive functioning (such as the ability to stay organized during a busy hospital shift, remember medication protocols, switch between patient cases quickly, and avoid emotional outbursts under pressure) and behavioral flexibility, are central to explaining who thrives under pressure and who succumbs to stress-related decline.

While previous research has documented the association between perceived stress and diminished quality of life (da Silva et al., 2024; Seo et al., 2018), few studies have examined the mediating role of self-regulation in this relationship, particularly in shift-working healthcare professionals. Understanding whether stress impairs quality of life via disruption to regulatory systems has both theoretical and applied significance. If confirmed, such a pathway would not only deepen insight into burnout vulnerability but also highlight self-regulation as a target for resilience-enhancing interventions in pharmacy and related health professions education and workforce development.

In line with educational and psychological models of human development, this study emphasizes both cognitive mechanisms and applied interventions relevant to training environments and professional transitions. Accordingly, the present study aims to determine whether executive self-regulation mediates the relationship between perceived stress and quality of life among shift-working healthcare professionals. In doing so, it bridges conceptual models of stress and resilience with empirical findings relevant to a high-risk occupational group. A secondary objective was to examine the reverse pathway, whether quality of life might improve stress levels through enhanced regulation. A third objective was to identify distinct behavioral clusters among participants based on their profiles of stress, self-regulation, and quality of life. Clarifying the directionality of these effects and uncovering unique phenotypes can inform precision interventions aimed at sustaining well-being in pharmacy and related health professions education, clinical training, and broader healthcare systems.

## 2. Materials and Methods

### 2.1. Participants and Procedure

This cross-sectional study was conducted between July 2024 and February 2025 among 82 shift-working healthcare and other academic professionals, recruited from clinical and academic settings at Notre Dame of Maryland University and affiliated rotation sites. A total of 135 individuals were initially approached via email and in-person recruitment. Of these, 82 provided consent and completed the study, yielding a participation rate of 60.7% and a rejection rate of 39.3%. Non-participation was mainly due to scheduling conflicts or lack of interest. The sample consisted of student pharmacists, pharmacy technicians and other healthcare professionals, all of whom routinely engaged in irregular or night shifts. Including these diverse professional roles allowed the study to capture a broad spectrum of shift-related stressors, regulatory challenges, and quality-of-life outcomes across both trainee and practicing healthcare worker populations. The survey was open to all shift-working healthcare and academic professionals at the institution and affiliated sites. Although participants included pharmacists, student pharmacists, pharmacy technicians, and other allied healthcare staff, the exact count for each role was not recorded. Eligible participants were full-time students or healthcare workers aged 18–60 years, fluent in English, and had engaged in at least one night or rotating shift (≥8 h) within the past 30 days. This criterion reflects prior evidence that the physiological and cognitive effects of shift work can persist up to 4–6 weeks post-exposure (American Academy of Sleep Medicine, 2014; Wickwire et al., 2017). Participants were excluded if they reported a current diagnosis of schizophrenia, major neurological disorder, or use of psychoactive medications known to impair cognitive function (e.g., antipsychotics, benzodiazepines).

Participants were categorized as active shift workers (currently engaged in shift work or exposure within the last 30 days during the study period). All participants provided informed consent, and the study protocol was approved by the Institutional Review Board at Notre Dame of Maryland University (IRB # PH043024SM01YMS).

### 2.2. Measures

- Perceived Stress Scale—4 item version (PSS-4):

The PSS-4 is a validated short-form measure assessing global stress appraisal over the past month using four Likert-type items, with higher scores indicating greater perceived stress (Cohen et al., 1983). The 4-item PSS demonstrated acceptable internal consistency in this sample (Cronbach’s α = 0.707).

- Executive Skills Questionnaire—Revised (ESQ-R):

The ESQ-R total score was used as a global index of self-regulation difficulty, with higher scores indicating greater impairment in executive functioning (Strait et al., 2020). All interpretations reflect this scoring direction. Although the ESQ-R includes five subscales (e.g., planning, working memory, emotional control, impulse regulation, and organization), only the total score was analyzed to capture overall self-regulatory capacity. The scale demonstrated excellent internal consistency in our sample (Cronbach’s α = 0.90), supporting its reliability in this context. Nevertheless, while ESQ-R is validated in educational settings, its sensitivity in occupational stress environments requires further evaluation.

- Quality of Life Scale (QOLS):

The 16-item QOLS assesses satisfaction across major life domains, including social, physical, psychological, and occupational well-being (Burckhardt & Anderson, 2003). Higher scores indicate better overall life quality. The QOLS exhibited excellent reliability in this cohort (Cronbach’s α = 0.91), indicating strong internal consistency in measuring overall quality of life.

Eighty-two shift-working healthcare professionals completed the study, offering a representative snapshot of healthcare trainees managing academic and occupational demands. The majority were in the 26–45 age range and female demographics that align with broader healthcare workforce trends. Nearly three-quarters reported working in urban settings, and approximately one-third had been exposed to a stable shift schedule for over two years, suggesting potential for cumulative circadian disruption. Most participants worked shifts of 8 h or less, and a substantial portion were single, reflecting transitional life stages often associated with increased psychosocial strain.

Mean scores on validated self-report instruments reflected moderate levels of stress (PSS-4: 6.49 ± 2.72) and executive self-regulation difficulty (ESQ-R: 21.91 ± 11.63). Despite these challenges, participants reported a relatively high quality of life (QOLS: 89.18 ± 14.40), with considerable inter-individual variability. These profiles form a data-rich foundation for modeling behavioral and psychological responses to shift work, insights that can be leveraged by AI systems designed for stress prediction, resilience monitoring, and digital phenotyping (see Table 1).

### 2.3. Statistical Analysis

Data was analyzed using SPSS v28.0. To test our main hypothesis, mediation analysis was performed using the PROCESS macro (Model 4) (Hayes, 2017). We examined whether executive self-regulation (ESQ-R) mediated the relationship between perceived stress (PSS) and quality of life (QOLS) and tested the reverse pathway (QOLS → PSS via ESQ-R). Indirect effects were estimated using bias-corrected bootstrapping with 5000 iterations, and significance was determined by 95% confidence intervals that did not include zero. Two mediation models were analyzed in this study dataset, i.e., a forward and a backward model is a case of multiple testing. So, a *p*-value adjustment (i.e., adjusted *p*-value = 0.05/2 (number of mediation models), and a confidence interval (CI) adjustment (i.e., changed *p*-value from 95% to 97.5% for bootstrap confidence intervals) were used to establish the significance level. To enhance the reproducibility and stability of our findings, we set the number of bootstrap iterations to 5126. This decision was based on preliminary analyses demonstrating that this number consistently yielded stable bias-corrected confidence intervals across repeated runs. This approach is consistent with methodological guidance in mediation analysis, which recommends using adequately large bootstrap samples to improve the precision, reliability, and replicability of indirect effect estimates (Hayes, 2017). To explore naturally occurring subgroups within the sample, unsupervised K-means clustering was conducted using standardized scores on perceived stress (PSS-4), self-regulation (ESQ-R), and quality of life (QOLS).

## 3. Results

### 3.1. Self-Regulation as a Mediator in the Effect of Perceived Stress on Quality of Life

This analysis evaluated the mediating effect of executive self-regulation on the relationship between perceived stress and quality of life among shift-working healthcare professionals.

Perceived stress (PSS) significantly and positively predicted executive self-regulation difficulties (b = 1.60, SE = 0.36, β = 0.44, *p* < 0.001, 95% CI = [0.78, 2.43]), suggesting that individuals experiencing greater stress reported more pronounced impairments in self-regulatory functioning.

In turn, higher self-regulation difficulty was significantly associated with reduced quality of life (b = −0.49, SE = 0.13, β = −0.40, *p* < 0.001, 95% CI = [−0.80, −0.19]), indicating that diminished regulatory control may play a critical role in deteriorating well-being.

The indirect effect of perceived stress on quality of life through self-regulation was statistically significant (b = −0.79, SE = 0.33, 95% CI = [−1.63, −0.17]), supporting indirect-only mediation, wherein the effect of stress on quality of life appears to operate primarily through regulatory disruption. Effect size analysis showed that self-regulation accounted for approximately 46% of the total effect in the forward mediation (stress → self-regulation → quality of life), suggesting a substantial mediating role.

The direct effect of stress on the quality of life was not statistically significant after accounting for the mediator (b = −0.94, SE = 0.48, β = −0.21, *p* = 0.054, 95% CI = [−2.03, 0.16]).

**Figure 1:**
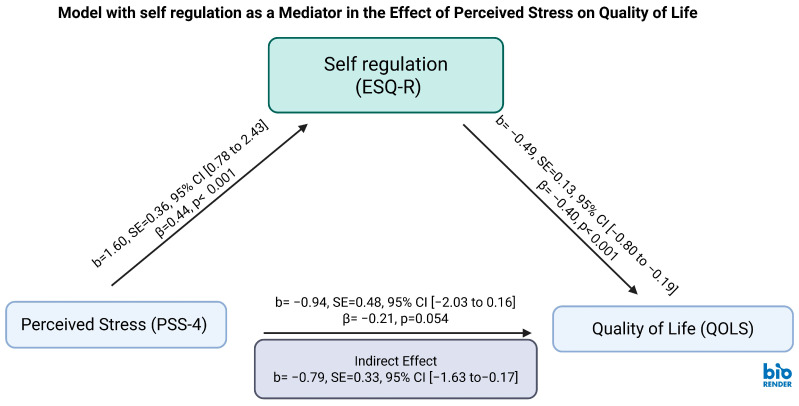
Forward Mediation Model of Stress, Self-Regulation, and Quality of Life. Mediation model testing the indirect effect of perceived stress on quality of life through self-regulation among shift-working healthcare professionals. PSS: Perceived Stress Scale; ESQ-R: Executive Skills Questionnaire–Revised; QOLS: Quality of Life Scale. Values shown are unstandardized coefficients (b) with standard errors (SE) and 95% confidence intervals (CI) in brackets. The indirect effect (b = −0.79, SE = 0.33, 95% CI = [−1.63, −0.17]) was statistically significant, supporting an indirect-only mediation model. Diagram created in BioRender Academic. Mohammed, S. (2025) https://BioRender.com/wei8jlo.

These findings underscore that higher levels of perceived stress impair executive self-regulation, which in turn compromises quality of life. The results highlight self-regulation as a central psychological pathway through which stress exerts its effects, aligning with an indirect-only mediation framework. (see Figure 1 and Table 2).

### 3.2. Self-Regulation Partially Mediates the Relationship Between Quality of Life and Perceived Stress

A reverse mediation analysis was conducted to examine whether self-regulation mediates the relationship between quality of life and perceived stress.

Quality of life (QOLS) was a statistically significant negative predictor of self-regulation difficulty (b = −0.40, SE = 0.08, β = −0.49, *p* < 0.001, 95% CI = [−0.58, −0.22]), suggesting that individuals with greater life satisfaction exhibited stronger executive self-regulation.

Self-regulation significantly predicted perceived stress in this model (b = −0.09, SE = 0.03, β = −0.34, *p* = 0.004, 95% CI = [−0.16, −0.02]), indicating that greater regulatory difficulty was associated with higher perceived stress levels.

**Figure 2:**
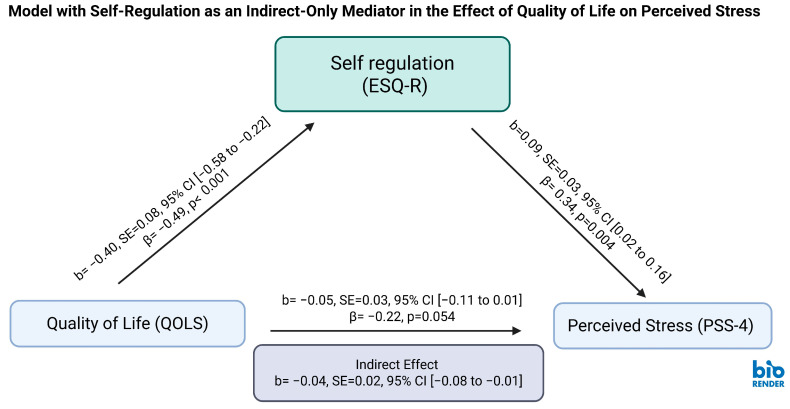
Reverse Mediation Model of Quality of Life, Self-Regulation, and Stress. Reverse mediation model testing whether self-regulation mediates the relationship between quality of life and perceived stress among shift-working healthcare professionals. QOLS: Quality of Life Scale; ESQ-R: Executive Skills Questionnaire–Revised; PSS: Perceived Stress Scale. The values shown are unstandardized coefficients (b) with standard errors in parentheses. The indirect effect was statistically significant (b = −0.04, 95% CI [−0.08, −0.01]), supporting an indirect-only mediation model. Created in BioRender Academic. Mohammed, S. (2025) https://BioRender.com/tsdcc1a.

The indirect effect of quality of life on perceived stress through self-regulation was statistically significant (b = −0.04, SE = 0.02, 95% CI = [−0.08, −0.01]), suggesting an indirect-only mediation model. Effect size analysis showed reverse pathway proportion accounted for 44% of the total effect, but its absolute effect size was small due to a weak total effect. The direct effect of quality of life on perceived stress was marginally significant (b = −0.05, SE = 0.03, β = −0.22, *p* = 0.054, 95% CI = [−0.11, 0.01]) (Figure 2; Table 2).

### 3.3. K-Means Analysis Identifies Behavioral Phenotypes Among Shift-Working Healthcare Professionals

A three-cluster solution provided a conceptually interpretable structure, identifying distinct behavioral profiles consistent with prior clustering literature (Wettstein et al., 2023; Table 3).

- Cluster 0 (“resilient cluster”) included participants with relatively high stress (M = 8.58) but moderate regulation difficulty (M = 23.73) and preserved QOL (M = 86.97).
- Cluster 1 (“low-strain cluster”) represented individuals with low stress (M = 3.84), low regulation difficulty (M = 15.00), and high QOL (M = 97.34).
- Cluster 2 (“high-strain cluster”) exhibited elevated stress (M = 9.36), severe regulation difficulty (M = 40.36), and markedly reduced QOL (M = 67.64).

These behavioral phenotypes may serve as a foundation for developing future screening tools and personalized wellness interventions in pharmacy and healthcare education settings.

## 4. Discussion

This study examined the mediating role of self-regulation in the relationship between perceived stress and quality of life among shift-working healthcare professionals. Mediation analysis revealed that higher stress levels significantly predicted self-regulatory impairments, which in turn were associated with diminished quality of life. Self-regulation significantly mediated both the forward and reverse pathways, though the effect was stronger in the stress-to-quality-of-life direction, supporting bidirectional but asymmetrical mediation. These findings indicate that nearly half of the relationship between stress and quality of life is explained by self-regulation, underscoring its pivotal role as a mediator. However, despite a similar proportion in the reverse model, its overall impact was negligible, confirming the directional dominance of the forward pathway. Together, these findings suggest that self-regulatory capacity is a key mechanism through which stress compromises well-being among professionals in high-demand, irregular work schedules. K-means clustering further supported this relationship by identifying distinct behavioral phenotypes defined by stress, self-regulation, and quality of life profiles. These findings underscore that higher levels of perceived stress appear to impair executive self-regulation, which in turn may compromise quality of life, aligning with an indirect-only mediation framework. Similarly, while a better quality of life was associated with reduced stress partly through improved self-regulation, this reverse pathway had weaker clinical relevance. Together, these observations reinforce self-regulation as a central psychological mechanism in stress-related impairment and highlight its potential as a target for resilience-enhancing interventions. While AI-enabled tools are not implemented in this study, our findings provide empirical input that could inform future development of predictive dashboards and digital phenotyping applications. These findings are consistent with established stress and resilience theories, supporting the role of regulatory processes in mediating the stress–quality of life link.

### 4.1. Self-Regulation as a Mediator Between Stress and Quality of Life

The forward mediation pathway observed in this study aligns with established stress and resilience frameworks, which emphasize that cognitive and emotional regulation capacities shape how individuals respond to stress (Gross, 1998; Lazarus & Folkman, 1984; McEwen, 1998). Recent evidence also shows that adaptive regulation strategies can mitigate burnout among healthcare professionals (Pálfi et al., 2024), underscoring the importance of targeting these skills in interventions. Our findings support this view, demonstrating that the indirect effect of stress on quality of life operates primarily through regulatory mechanisms, while the reverse pathway is weaker, highlighting an asymmetrical but bidirectional relationship.

Our findings add empirical support to this view in the context of shift-working healthcare professionals, who face compounding risks such as exposure to bright light, sleep deprivation, circadian disruption, and excessive caffeine consumption (Kalmbach et al., 2018; Salahuddin et al., 2025a; Soleimani et al., 2024; Wickwire et al., 2017; Zeek et al., 2015). These stressors can impair prefrontal cortex functions responsible for executive control, while simultaneously heightening amygdala reactivity and dysregulating hypothalamic–pituitary–adrenal (HPA) axis function, mechanisms that degrade working memory, emotion regulation, and behavioral inhibition (Kinlein et al., 2015; McEwen & Morrison, 2013). Our data support the hypothesis that these impairments are not merely side effects of stress but active mediators of its impact on quality of life, underscoring the neurocognitive cost of chronic stress exposure in this population.

Building on our earlier findings related to sex differences in stress biomarkers among student pharmacists (Salahuddin et al., 2025a, 2025b), this study further addresses a critical gap in the literature concerning shift-working healthcare professionals. While prior research has shown that pharmacy students experience high stress levels, poor sleep quality, and frequent stimulant use (Zeek et al., 2015; Cates et al., 2015; Hagemann et al., 2020), most studies in this space have focused on descriptive statistics or direct correlations, rather than underlying explanatory mechanisms. Furthermore, burnout interventions in healthcare training have tended to emphasize mindfulness and stress awareness, while often overlooking executive functioning and behavioral regulation skills that may buffer against real-time decision fatigue, emotional overload, and performance lapses (Rompilla et al., 2023). By centering self-regulation as a mediating mechanism and assessing it via the ESQ-R, this study adds theoretical depth and practical utility to the conversation on pharmacy and related health professions education and clinical workforce resilience (Langran et al., 2022; Murry & Witry, 2020). It also lays conceptual groundwork for integrating digital monitoring tools that can track self-regulatory breakdowns and predict quality-of-life outcomes. (Oudin et al., 2023).

The findings point to clear educational and institutional implications. As burnout and stress-related impairment rise among healthcare trainees and practitioners (Batanda, 2024), efforts to promote resilience must move beyond self-care slogans and toward evidence-based interventions that strengthen regulatory capacity (Luthar & Cicchetti, 2000). Training modules in cognitive reappraisal, flexible goal-setting, and impulse control, key subdomains of self-regulation, can be integrated into pharmacy and related health profession curricula, residency onboarding, and continuing education (Cutuli, 2014; Deci & Ryan, 2000). These skills are particularly important during transitional periods when regulatory demands are high and cognitive load increases (Luciana, 2016). Furthermore, brief executive functioning screeners may help identify at-risk individuals during times of transition (e.g., final year placements and shift rotations; Johnson et al., 2007). Tailoring interventions to support those with high cognitive load and poor sleep, especially shift-working healthcare professionals, may offer a cost-effective way to prevent burnout and promote quality of care (Hagemann et al., 2020; Razai et al., 2023).

### 4.2. Behavioral Profiles Support Personalized Intervention Targets

To further investigate the heterogeneity of experiences within the sample, unsupervised K-means clustering revealed three distinct stress–regulation–well-being profiles. These included a “resilient cluster” group with high stress, moderate regulation difficulty, and preserved quality of life; a “low-strain cluster” group with low stress, low regulation difficulty, and high quality of life; and a “high-strain cluster” group marked by high stress, high regulation difficulty, and diminished QOL. Prior studies have shown that data-driven profiling using clustering methods can identify meaningful psychological subtypes across stress, coping, and mental health dimensions (Drysdale et al., 2017; Hudon et al., 2023; Wang et al., 2021). These findings provide empirical support for the development of AI-enabled screening tools that could classify frontline professionals into high- and low-risk categories, supporting early identification of burnout trajectories (Ghosh et al., 2022; Kamath et al., 2022; Lazarou & Exarchos, 2024). Clustering also aligns with precision mental health frameworks, suggesting that resilience interventions may benefit from stratification based on behavioral phenotypes rather than assuming a one-size-fits-all model (Insel, 2014; Kapur et al., 2012; Mohr et al., 2017).

The present findings highlight the utility of these clusters in guiding tailored intervention strategies. For instance, individuals in the low-stress, high-regulation, high-QOL cluster may require minimal intervention, focusing instead on maintenance and periodic well-being monitoring. In contrast, those in the burnout-risk cluster, with elevated stress and compromised self-regulation, may benefit from intensive support such as cognitive-behavioral therapy, stress inoculation training, or digital mind–body interventions. Intermediate profiles, such as a resilient cluster, could benefit from resilience training to maintain function under continued stress exposure. Therefore, the cluster-based approach not only enhances conceptual understanding of stress vulnerability but also offers a practical framework for optimizing resource allocation and intervention delivery, saving time, reducing cost, and improving outcomes through targeted mental health support.

### 4.3. Quality of Life Exhibits a Partial Indirect Effect on Stress via Self-Regulation

The reverse mediation model, in which quality of life was hypothesized to improve stress levels via stronger self-regulation, was partially supported. While higher life satisfaction was modestly associated with better regulatory capacity, this improvement did not translate to reduced perceived stress, supporting an indirect-only pathway with a marginal direct effect. This aligns with resource-based theories such as the Strength Model of Self-Control (Baumeister et al., 2007) and Ego Depletion Theory (Baumeister et al., 1998), which suggest that regulation is more often depleted by stress than bolstered by well-being. Neurobiological evidence also shows that chronic stress impairs the very neural circuits involved in control, meaning that top-down recovery often requires specific intervention, not just environmental or hedonic improvements (McEwen & Morrison, 2013; Mika et al., 2012; Wolff et al., 2021). In healthcare, this reinforces that even satisfied professionals may not be shielded from stress-related cognitive breakdown unless they are actively engaging in regulation-enhancing strategies.

### 4.4. Limitations

Nonetheless, several limitations must be noted. First, the cross-sectional design restricts causal interpretation, although the model aligns with prior longitudinal frameworks. Notably, while both mediation models yielded statistically significant indirect effects, the small sample size may limit the stability of these findings and inflate the risk of Type I and II errors. These results do not appear to be explained by multicollinearity, suppression effects, or model misspecification. Future work should use larger sample sizes and temporal or experimental designs to validate these pathways. Additionally, digital health approaches such as wearable sensors or mobile-based behavioral analytics may offer scalable methods to monitor regulatory functions in real time. Second, reliance on self-report instruments may introduce bias; incorporating physiological (e.g., cortisol), behavioral (e.g., cognitive tasks), or passive sensing data (e.g., actigraphy) could improve measurement accuracy and reduce self-report bias. Third, the sample was limited to shift-working healthcare professionals, potentially reducing generalizability to daytime workers or those in less cognitively demanding occupations. The moderate response rate (60.7%) and non-random recruitment process may have introduced selection bias, limiting generalizability. Additionally, the absence of detailed counts for specific occupational roles limits the ability to explore role-specific differences in stress, regulation, and quality-of-life outcomes. Furthermore, the small sample size, predominantly urban and female composition, and reliance on voluntary participation constrain the representativeness of findings. Future research should include more diverse and larger samples to confirm these results. Moreover, while descriptive data on shift length were collected, subgroup analyses comparing different shift durations (e.g., <8 h vs. ≥12 h) were not conducted. Shift exposure intensity may influence stress and regulation outcomes, and future studies should investigate whether variations in shift duration alter these relationships. Finally, unmeasured contextual factors such as job control, social support, and organizational culture may moderate the observed effects and should be examined in future studies.

## 5. Conclusions

In conclusion, this study identifies self-regulation as a core cognitive-affective mechanism mediating the relationship between perceived stress and quality of life in shift-working healthcare professionals. These findings reflect an indirect-only mediation model in both forward and reverse directions, with stress impairing quality of life through regulatory difficulty, and quality of life likewise exerting an effect on stress through improved regulation, though to a lesser extent. This suggests that strengthening executive functioning may serve as a high-yield target for bolstering occupational resilience, especially when direct reductions in stress exposure are not feasible. Supported by theoretical and biological models, the findings emphasize the mediating role of self-regulation in translating stress into diminished well-being, reinforcing the need for cognitive-affective intervention strategies. These insights point toward scalable, theory-driven interventions in pharmacy education and health systems aimed at strengthening executive control and promoting resilience in one of the most essential and at-risk segments of the healthcare workforce. Incorporating such models into future digital health tools or predictive dashboards may help support workforce resilience initiatives. While AI was not applied in this study, the identified behavioral phenotypes and mediation pathways provide an empirical basis that future digital health technologies may leverage to enhance precision in burnout prevention strategies. Using only the ESQ-R total score may obscure domain-specific patterns (e.g., which executive functions are most affected by stress). Moreover, although reliability in this sample was high (α = 0.90), future research should further validate the ESQ-R in occupational stress contexts to ensure its predictive utility for healthcare workers. Embedding self-regulation training into pharmacy and related health profession education and institutional wellness strategies may improve resilience, reduce burnout, and better equip professionals for the demands of shift-based care.

## Figures and Tables

**Table 1:** Participants’ Characteristics.

Characteristics	Mean ± SD/ Frequency (Percentage)
Age (years)	18–25: 32 (39%)
	26–45: 42 (51.2%)
	>45: 8 (9.8%)
Gender	Male: 21 (25.6%)
	Female: 61 (74.4%)
Residence	City: 76 (92.7%)
	Countryside: 6 (7.3%)
Marital Status	Single: 66 (80.5%)
	Married: 14 (17.1%)
	Divorced/Separated: 2 (2.4%)
Shift Hours Per Day	Less than or equal to 8 h: 44 (53.7%)
	8–12 h:35 (42.6%)
	Greater than 12 h: 3 (3.7%)
How long have you been working in the same shift?	1–6 months: 16 (19.5%)
	6–12 months: 22 (26.8%)
	Greater than 12 months: 18 (22%)
	Greater than 24 months: 26 (31.7%)
PSS -4 (Perceived Stress Score)	6.49 ± 2.72
ESQ- R Scores (Executive Skills Questionnaire–revised)	21.91 ± 11.63
QOLS (Quality of Life Score)	89.18 ± 14.40

Note: Some variables in this table were previously analyzed in a separate study; they are included here as part of a new investigation exploring a different hypothesis.

**Table 2:** Summary of Mediation and Reverse Mediation Models for Stress, Self-Regulation, and Quality of Life.

Pathway	Total Effect (b)	Direct Effect (b)	Indirect Effect (b)	95% CI (Indirect Effect)	Type of Mediation Model
Stress → Self-Regulation → Quality of Life (Forward)	−1.73 *	−0.94	−0.79 *	[−1.63, −0.17]	Indirect-only mediation
Quality of Life → Self-Regulation → Stress (Reverse)	−0.09 *	−0.05	−0.04 *	[−0.08, −0.01]	Indirect-only mediation

* significant at *p* = 0.025. Note: Values represent unstandardized coefficients (b). Confidence intervals are based on 5126 bootstrapped samples.

**Table 3:** Cluster-Derived Behavioral Profiles Based on Stress, Self-Regulation, and Quality of Life.

Cluster	PSS (Stress) Mean ± SD	ESQ-R (Regulation Difficulty) Mean ± SD	QOL (Quality of Life) Mean ± SD	Interpretation
0	8.58 ± 2.11	23.73 ± 8.71	86.97 ± 11.53	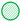 High stress, moderate regulation difficulty, but high QOL—resilient cluster
1	3.84 ± 2.06	15.00 ± 6.70	97.34 ± 10.18	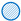 Low stress, low regulation difficulty, high QOL—low-strain cluster
2	9.36 ± 1.96	40.36 ± 10.93	67.64 ± 9.55	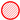 High stress, high regulation difficulty, low QOL—high-strain cluster

Note: All the 3 clusters have significant differences across all the dependent variables (Stress, Regulation difficulty, and Quality of Life).

## Data Availability

The authors confirm that the data supporting the findings of this study are available within the article as Supplementary Materials.

## Supplementary Materials

The following supporting information can be downloaded at: https://www.mdpi.com/article/10.3390/ejihpe15090180/s1.

## References

[B1-ejihpe-15-00180] American Academy of Sleep Medicine (2014). International classification of sleep disorders: Diagnostic and coding manual.

[B2-ejihpe-15-00180] Bakker A. B., Demerouti E. (2017). Job demands-resources theory: Taking stock and looking forward. Journal of Occupational Health Psychology.

[B3-ejihpe-15-00180] Barnett I., Torous J., Staples P., Sandoval L., Keshavan M., Onnela J. P. (2018). Relapse prediction in schizophrenia through digital phenotyping: A pilot study. Neuropsychopharmacology.

[B4-ejihpe-15-00180] Batanda I. (2024). Prevalence of burnout among healthcare professionals: A survey at fort portal regional referral hospital. NPJ Mental Health Research.

[B5-ejihpe-15-00180] Baumeister R. F., Bratslavsky E., Muraven M., Tice D. M. (1998). Ego depletion: Is the active self a limited resource?. Journal of Personality and Social Psychology.

[B6-ejihpe-15-00180] Baumeister R. F., Vohs K. D., Tice D. M. (2007). The strength model of self-control. Current Directions in Psychological Science.

[B7-ejihpe-15-00180] Billore S., Anisimova T., Vrontis D. (2023). Self-regulation and goal-directed behavior: A systematic literature review, public policy recommendations, and research agenda. Journal of Business Research.

[B8-ejihpe-15-00180] Burckhardt C. S., Anderson K. L. (2003). The Quality of Life Scale (QOLS): Reliability, validity, and utilization. Health and Quality of Life Outcomes.

[B9-ejihpe-15-00180] Cates M. E., Clark A., Woolley T. W., Saunders A. (2015). Sleep quality among pharmacy students. American Journal of Pharmaceutical Education.

[B10-ejihpe-15-00180] Cohen S., Kamarck T., Mermelstein R. (1983). A global measure of perceived stress. Journal of Health and Social Behavior.

[B11-ejihpe-15-00180] Cutuli D. (2014). Cognitive reappraisal and expressive suppression strategies role in the emotion regulation: An overview on their modulatory effects and neural correlates. Frontiers in Systems Neuroscience.

[B12-ejihpe-15-00180] da Silva C. C. M., Santos A. B. D., Leoci I. C., Leite E. G., Antunes E. P., Torres W., Mesquita E. D. L., Delfino L. D., Beretta V. S. (2024). The association between perceived stress, quality of life, and level of physical activity in public school teachers. International Journal of Environmental Research and Public Health.

[B13-ejihpe-15-00180] Dawson P., Guare R. (2016). The smart but scattered guide to success: How to use your brain’s executive skills to keep up, stay calm, and get organized at work and at home.

[B14-ejihpe-15-00180] Deci E. L., Ryan R. M. (2000). The “what” and “why” of goal pursuits: Human needs and the self-determination of behavior. Psychological Inquiry.

[B15-ejihpe-15-00180] Drysdale A. T., Grosenick L., Downar J., Dunlop K., Mansouri F., Meng Y., Fetcho R. N., Zebley B., Oathes D. J., Etkin A., Schatzberg A. F., Sudheimer K., Keller J., Mayberg H. S., Gunning F. M., Alexopoulos G. S., Fox M. D., Pascual-Leone A., Voss H. U., Liston C. (2017). Resting-state connectivity biomarkers define neurophysiological subtypes of depression. Nature Medicine.

[B16-ejihpe-15-00180] Ghosh S., Kim S., Ijaz M. F., Singh P. K., Mahmud M. (2022). Classification of mental stress from wearable physiological sensors using image-encoding-based deep neural network. Biosensors.

[B17-ejihpe-15-00180] Gross J. J. (1998). The emerging field of emotion regulation: An integrative review. Review of General Psychology.

[B18-ejihpe-15-00180] Hagemann T. M., Reed B. N., Bradley B. A., Clements J. N., Cohen L. J., Coon S. A., Derington C. G., DiScala S. L., El-Ibiary S., Lee K. C., May A., Oh S., Phillips J. A., Rogers K. M. (2020). Burnout among clinical pharmacists: Causes, interventions, and a call to action. Journal of the American College of Clinical Pharmacy.

[B19-ejihpe-15-00180] Hayes A. F. (2017). Partial, conditional, and moderated moderated mediation: Quantification, inference, and interpretation. Communication Monographs.

[B20-ejihpe-15-00180] Heatherton T. F. (2011). Neuroscience of self and self-regulation. Annual Review of Psychology.

[B21-ejihpe-15-00180] Huckvale K., Venkatesh S., Christensen H. (2019). Toward clinical digital phenotyping: A timely opportunity to consider purpose, quality, and safety. NPJ Digital Medicine.

[B22-ejihpe-15-00180] Hudon A., Beaudoin M., Phraxayavong K., Potvin S., Dumais A. (2023). Unsupervised machine learning driven analysis of verbatims of treatment-resistant schizophrenia patients having followed avatar therapy. Journal of Personalized Medicine.

[B23-ejihpe-15-00180] Insel T. R. (2014). The NIMH Research Domain Criteria (RDoC) Project: Precision medicine for psychiatry. American Journal of Psychiatry.

[B24-ejihpe-15-00180] Jehan S., Zizi F., Pandi-Perumal S. R., Myers A. K., Auguste E., Jean-Louis G., McFarlane S. I. (2017). Shift work and sleep: Medical implications and management. Sleep Medicine and Disorders: International Journal.

[B25-ejihpe-15-00180] Johnson J. K., Lui L. Y., Yaffe K. (2007). Executive function, more than global cognition, predicts functional decline and mortality in elderly women. Journal of Gerontology: Series A, Biological Sciences and Medical Sciences.

[B26-ejihpe-15-00180] Kadović M., Mikšić Š., Lovrić R. (2022). Ability of emotional regulation and control as a stress predictor in healthcare professionals. International Journal of Environmental Research and Public Health.

[B27-ejihpe-15-00180] Kalisch R., Müller M. B., Tüscher O. (2015). A conceptual framework for the neurobiological study of resilience. Behavioral and Brain Sciences.

[B28-ejihpe-15-00180] Kalmbach D. A., Anderson J. R., Drake C. L. (2018). The impact of stress on sleep: Pathogenic sleep reactivity as a vulnerability to insomnia and circadian disorders. Journal of Sleep Research.

[B29-ejihpe-15-00180] Kamath J., Leon Barriera R., Jain N., Keisari E., Wang B. (2022). Digital phenotyping in depression diagnostics: Integrating psychiatric and engineering perspectives. World Journal of Psychiatry.

[B30-ejihpe-15-00180] Kapur S., Phillips A. G., Insel T. R. (2012). Why has it taken so long for biological psychiatry to develop clinical tests and what to do about it?. Molecular Psychiatry.

[B31-ejihpe-15-00180] Khan W. A. A., Jackson M. L., Kennedy G. A., Conduit R. (2021). A field investigation of the relationship between rotating shifts, sleep, mental health and physical activity of Australian paramedics. Scientific Reports.

[B32-ejihpe-15-00180] Kinlein S. A., Wilson C. D., Karatsoreos I. N. (2015). Dysregulated hypothalamic-pituitary-adrenal axis function contributes to altered endocrine and neurobehavioral responses to acute stress. Frontiers in Psychiatry.

[B33-ejihpe-15-00180] Langran C., Mantzourani E., Hughes L., Hall K., Willis S. (2022). “I’m at breaking point”: Exploring pharmacists’ resilience, coping and burnout during the COVID-19 pandemic. Exploratory Research in Clinical and Social Pharmacy.

[B34-ejihpe-15-00180] Lazarou E., Exarchos T. P. (2024). Predicting stress levels using physiological data: Real-time stress prediction models utilizing wearable devices. AIMS Neuroscience.

[B35-ejihpe-15-00180] Lazarus R. S., Folkman S. (1984). Stress, appraisal and coping.

[B36-ejihpe-15-00180] Lee Y., Ragguett R.-M., Mansur R. B., Boutilier J. J., Rosenblat J. D., Trevizol A., Brietzke E., Lin K., Pan Z., Subramaniapillai M., Chan T. C. Y., Fus D., Park C., Musial N., Zuckerman H., Chen V. C.-H., Ho R., Rong C., McIntyre R. S. (2018). Applications of machine learning algorithms to predict therapeutic outcomes in depression: A meta-analysis and systematic review. Journal of Affective Disorders.

[B37-ejihpe-15-00180] Lin L., Zhang X., Wang P. (2025). Interconnected stressors and well-being in healthcare professionals. Applied Research in Quality of Life.

[B38-ejihpe-15-00180] Luciana M. (2016). Executive function in adolescence: A commentary on regulatory control and depression in adolescents: Findings from neuroimaging and neuropsychological research. Journal of Clinical Child and Adolescent Psychology.

[B39-ejihpe-15-00180] Luthar S. S., Cicchetti D. (2000). The construct of resilience: Implications for interventions and social policies. Development and Psychopathology.

[B40-ejihpe-15-00180] McEwen B. S. (1998). Stress, adaptation, and disease. Allostasis and allostatic load. Annals of the New York Academy of Sciences.

[B41-ejihpe-15-00180] McEwen B. S., Morrison J. H. (2013). The brain on stress: Vulnerability and plasticity of the prefrontal cortex over the life course. Neuron.

[B42-ejihpe-15-00180] Mika A., Mazur G. J., Hoffman A. N., Talboom J. S., Bimonte-Nelson H. A., Sanabria F., Conrad C. D. (2012). Chronic stress impairs prefrontal cortex-dependent response inhibition and spatial working memory. Behavioral Neuroscience.

[B43-ejihpe-15-00180] Mohr D. C., Zhang M., Schueller S. M. (2017). Personal sensing: Understanding mental health using ubiquitous sensors and machine learning. Annual Review of Clinical Psychology.

[B44-ejihpe-15-00180] Murry L. T., Witry M. J. (2020). ‘Wasting time inside my mind’: Exploring student pharmacists’ perspectives on engaging in mindfulness oriented meditation using concepts from education research. Pharmacy Education.

[B45-ejihpe-15-00180] Oudin A., Maatoug R., Bourla A., Ferreri F., Bonnot O., Millet B., Schoeller F., Mouchabac S., Adrien V. (2023). Digital phenotyping: Data-driven psychiatry to redefine mental health. Journal of Medical Internet Research.

[B46-ejihpe-15-00180] Pálfi K., Major J., Horváth-Sarródi A., Deák A., Fehér G., Gács B. (2024). Adaptive emotion regulation might prevent burnout in emergency healthcare professionals: An exploratory study. BMC Public Health.

[B47-ejihpe-15-00180] Razai M. S., Kooner P., Majeed A. (2023). Strategies and interventions to improve healthcare professionals’ well-being and reduce burnout. Journal of Primary Care & Community Health.

[B48-ejihpe-15-00180] Rech M. A., Jones G. M., Naseman R. W., Beavers C. (2022). Premature attrition of clinical pharmacists: Call to attention, action, and potential solutions. Journal of the American College of Clinical Pharmacy.

[B49-ejihpe-15-00180] Rompilla D. B., Stephens J. E., Martinez M., Mikels J. A., Haase C. M. (2023). Can emotional acceptance buffer the link between executive functioning and mental health in late life?. Emotion.

[B50-ejihpe-15-00180] Salahuddin M. F., Bugingo R., Mahdi F., Spencer D., Manzar M. D., Paris J. J. (2025a). Physiological and psychological impacts of shift work among student pharmacists: Sex differences in stress and health outcomes. Psychiatry International.

[B51-ejihpe-15-00180] Salahuddin M. F., Samuel B. I., Bugingo R., Spencer D., Manzar M. D., BaHammam A. S. (2025b). The mediating role of negative mood affect in the relationship between perceived stress and vulnerability to insomnia among student pharmacist shift workers. Nature and Science of Sleep.

[B52-ejihpe-15-00180] Schommer J. C., Gaither C. A., Alvarez N. A., Lee S., Shaughnessy A. M., Arya V., Planas L. G., Fadare O., Witry M. J. (2022). Pharmacy workplace wellbeing and resilience: Themes identified from a hermeneutic phenomenological analysis with future recommendations. Pharmacy.

[B53-ejihpe-15-00180] Seo E. J., Ahn J. A., Hayman L. L., Kim C. J. (2018). The association between perceived stress and quality of life in university students: The parallel mediating role of depressive symptoms and health-promoting behaviors. Asian Nursing Research.

[B54-ejihpe-15-00180] Soleimani E., Tahmasebi R., Daneshmandi H., Salimi S. H., Aliasghari F. (2024). Work-life balance and health among pharmacists: Physical activity, sleep quality, and general health. BMC Health Services Research.

[B55-ejihpe-15-00180] Strait J. E., Dawson P., Walther C. A. P., Strait G. G., Barton A. K., Brunson McClain M. (2020). Refinement and psychometric evaluation of the executive skills questionnaire-revised. Contemporary School Psychology.

[B56-ejihpe-15-00180] Ugwu L. E., Idemudia E. S., Onyedibe M. C. C. (2024). Decoding the impact of night/day shiftwork on well-being among healthcare workers. Scientific Reports.

[B57-ejihpe-15-00180] Walker W. H., Walton J. C., DeVries A. C., Nelson R. J. (2020). Circadian rhythm disruption and mental health. Translational Psychiatry.

[B58-ejihpe-15-00180] Wang Y., Tang S., Zhang L., Bu X., Lu L., Li H., Gao Y., Hu X., Kuang W., Jia Z., Sweeney J. A., Gong Q., Huang X. (2021). Data-driven clustering differentiates subtypes of major depressive disorder with distinct brain connectivity and symptom features. British Journal of Psychiatry.

[B59-ejihpe-15-00180] Wettstein A., Jenni G., Schneider I., Kühne F., Grosse Holtforth M., La Marca R. (2023). Predictors of psychological strain and allostatic load in teachers: Examining the long-term effects of biopsychosocial risk and protective factors using a LASSO regression approach. International Journal of Environmental Research and Public Health.

[B60-ejihpe-15-00180] Wickwire E. M., Geiger-Brown J., Scharf S. M., Drake C. L. (2017). Shift work and shift work sleep disorder: Clinical and organizational perspectives. Chest.

[B61-ejihpe-15-00180] Wolff M., Enge S., Kräplin A., Krönke K.-M., Bühringer G., Smolka M. N., Goschke T. (2021). Chronic stress, executive functioning, and real-life self-control: An experience sampling study. Journal of Personality.

[B62-ejihpe-15-00180] Zeek M. L., Savoie M. J., Song M., Kennemur L. M., Qian J., Jungnickel P. W., Westrick S. C. (2015). Sleep duration and academic performance among student pharmacists. American Journal of Pharmaceutical Education.

